# Association of red blood cell distribution width and hemoglobin‐to‐RDW ratio with contrast‐associated acute kidney injury in patients undergoing coronary angiography: A retrospective study

**DOI:** 10.1002/clc.24163

**Published:** 2023-10-04

**Authors:** Lijie Zhu, Zhezhe Chen, Hangpan Jiang, Peng Wang, Tianli Hu, Menghan Gao, Xiaolong Hu, Maoning Lin, Xianglan Liu, Wenbin Zhang

**Affiliations:** ^1^ Department of Cardiology, Sir Run Run Shaw Hospital, College of Medicine Zhejiang University Hangzhou Zhejiang China; ^2^ Key Laboratory of Cardiovascular Intervention and Regenerative Medicine of Zhejiang Province Hangzhou Zhejiang China; ^3^ Department of Cardiology, The Fourth Affiliated Hospital, College of Medicine Zhejiang University Yiwu Zhejiang China; ^4^ Department of Cardiology, College of Medicine Zhejiang University Hangzhou Zhejiang China

**Keywords:** contrast‐associated acute kidney injury, coronary angiography, hemoglobin, inflammation, red blood cell distribution width

## Abstract

**Background:**

Inflammation contributes to poor prognosis in cardiovascular diseases. A novel biomarker for systemic inflammation that has garnered attention is the red blood cell distribution width (RDW). This study is designed to explore potential associations between RDW and hemoglobin‐to‐RDW ratio (HRR) with contrast‐associated acute kidney injury (CA‐AKI).

**Methods:**

This study retrospectively analyzed 4054 patients undergoing coronary angiography (CAG). Linear regression models were employed to assess the relationships between RDW or HRR and the elevation of serum creatinine (Scr). The associations between RDW or HRR and CA‐AKI were explored using restricted cubic spline and log‐binomial regression analyses taking into account specific cutoff values and quintiles. Exploratory analyses were also conducted to further investigate these associations.

**Results:**

Among enrolled patients, the average age was 66.9 years and 34.3% were female. Notably, patients who developed CA‐AKI tended to have higher RDW and lower HRR. Multivariable linear regression models demonstrated that RDW exhibited a positive association with Scr elevation (*β* = 2.496, 95% confidence interval [CI] = 1.784–3.208), while HRR displayed a negative association (*β* = −3.559, 95% CI = −4.243 to −2.875). Multivariable log‐binomial regression models confirmed that both high RDW (RDW ≥ 13.8%) and low HRR (HRR < 8.9) were significantly associated with a higher risk of CA‐AKI (RDW [≥13.8% vs. <13.8%]: relative risk [RR] = 1.540, 95% CI = 1.345–1.762; HRR [<8.9 vs. ≥8.9]: RR = 1.822, 95% CI = 1.584–2.096). Exploratory analysis determined that such associations still existed regardless of age, gender, estimated glomerular filtration rate, or anemia.

**Conclusions:**

Elevated preoperative RDW and decreased HRR were significantly associated with CA‐AKI in patients undergoing CAG.

## Graphical abstract

Both high red blood cell distribution width (RDW) and low hemoglobin‐to‐RDW ratio (HRR) demonstrated robust and independent associations with contrast‐associated acute kidney injury (CA‐AKI). These findings suggest that tracking RDW and HRR could be valuable for the early identification of CA‐AKI risk.

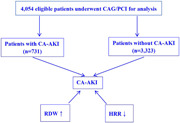

## 1 INTRODUCTION

Contrast‐associated acute kidney injury (CA‐AKI) is a significant concern in medical practice, ranking as the third leading cause of hospital‐acquired acute kidney injury.^1^ Its prevalence varies, affecting 1%–2% of the general population, but the risk can be significantly higher, up to 50%, in high‐risk groups.^2^, ^3^ Several established risk factors, including severe chronic kidney disease, hypotension, excessive contrast medium, anemia, and diabetes, have been linked to an increased likelihood of CA‐AKI and have been incorporated into risk stratification models.^1^, ^4^

Inflammation has been identified as a key player in the development and progression of various diseases, including cardiovascular conditions like CA‐AKI.^5^ Numerous studies have highlighted the prognostic value of systemic inflammatory markers in CA‐AKI patients, independent of traditional risk factors. These markers include C‐reactive protein (CRP), interleukin‐6, and white blood cell count.^6^, ^7^

In addition to established inflammatory markers, red blood cell distribution width (RDW) has gained attention as an informative and cost‐effective indicator of systemic inflammation.^8^, ^9^ A growing body of research has demonstrated the prognostic significance of RDW in inflammation‐related cardiovascular diseases, such as atherosclerosis and ischemic cardiac conditions.^10^, ^11^, ^12^, ^13^ Hemoglobin, on the other hand, is a critical parameter reflecting anemia, which is a traditional risk factor for CA‐AKI. Hemoglobin‐to‐RDW ratio (HRR), a novel metric introduced by Sun et al., is a novel indicator of chronic inflammation combining the prognostic information from hemoglobin and RDW.^14^ Accordingly, the accessibility of RDW and HRR in clinical practice for CA‐AKI seems reasonable and potentially valuable.

Therefore, this retrospective study set out to determine the associations of RDW and HRR with CA‐AKI in patients undergoing coronary angiography (CAG) or percutaneous coronary intervention (PCI), for the sake of aggressive management to reduce the risk of CA‐AKI.

## 2 METHODS

### 2.1 Study population

This study was designed as a retrospective and observational investigation, focusing on patients who underwent CAG or PCI at Sir Run Run Shaw Hospital between December 2009 and December 2019. Supporting Information: Figure S1 illustrates the step‐by‐step process for patient selection. Patients meeting the following criteria were included: (1) complete data available regarding their demographic characteristics and laboratory test results, and (2) serum creatinine (Scr) measurements obtained both at baseline and within a 72‐hour window following the administration of contrast media. Patients were excluded if they met any of the following criteria: (1) received multiple contrast media administrations during their hospital stay, (2) had a preprocedure estimated glomerular filtration rate (eGFR) below 15 mL/min/1.73 m², (3) were diagnosed with severe cardiac insufficiency, active malignant tumors, or autoimmune diseases, or (4) passed away within 1 month after undergoing PCI. The study was conducted in accordance with the Declaration of Helsinki and ethical approval was granted from the Ethics Committee of Sir Run Run Shaw Hospital (No. 20201217‐36). Furthermore, the research adhered to the Strengthening the Reporting of Observational Studies in Epidemiology guidelines.^15^ As a retrospective study, informed consent was not required.

### 2.2 Sample size estimation

The sample size for this study was determined following a common rule of thumb,^16^ which suggests having a minimum of 20 patients with the condition of interest (in this case, CA‐AKI) for each variable being studied. In this study, nine potential risk factors were considered. Previous studies have indicated that the incidence of CA‐AKI fluctuated mainly between 5% and 20%.^17^, ^18^, ^19^ Therefore, at least 180 patients with CA‐AKI and 3600 patients undergoing CAG or PCI were required.

### 2.3 Data collection

Clinical data for the enrolled patients were collected from the Hospital Information System. Fasting venous blood samples were collected for hematological and biochemical tests. Scr was measured at baseline and more than three times within 72 hours following administration of contrast media. RDW was automatically analyzed by the automatic hematology analyzer and blood cell counter (XE‐2100; Sysmex) and was recorded as the coefficient of variation in inspection reports.^20^ The normal reference range for RDW at our hospital is 11.0%–14.5%. The normal reference range for hemoglobin at our hospital is 130–175 g/L. The Chronic Kidney Disease Epidemiology Collaboration equation was utilized to calculate eGFR, which is based on Scr at baseline, age, sex, and race.^21^

### 2.4 Definitions

The diagnosis of CA‐AKI was made following the criteria set by the European Society of Urogenital Radiology. CA‐AKI was diagnosed if there was either: (1) An increase in Scr levels of more than 0.5 mg/dL (44.2 μmol/L) and (2) an increase in the Scr ratio of more than 25%.^22^ Scr elevation was calculated using the following formula: Scr elevation = (Maximum postoperative Scr within 72 hours – Scr at baseline)/Scr at baseline × 100%. HRR was calculated as hemoglobin (g/L) divided by RDW (%). The recommended maximum dose of contrast media was calculated using the following formula: 5 mL × weight (kg)/Scr at baseline (mg/dL).^23^ Excessive contrast media was defined as the actual dose of contrast media administered exceeding the recommended maximum dose.

### 2.5 Statistical analysis

Continuous variables were expressed as mean and standard deviation or median (interquartile range) according to distribution. Intergroup comparisons were conducted by using Student's *t* test for normally distributed variables, and the Mann–Whitney *U* test if not. Categorical variables were presented as numbers and proportions, and the *χ* ^2^ test was performed for comparisons between groups.

The receiver operating characteristic (ROC) curve was performed to determine the cutoff values of RDW and HRR. Linear regression analysis was performed to explore the associations between RDW or HRR and Scr elevation. The multivariable analysis incorporated recognized risk factors for CA‐AKI.^24^, ^25^ Restricted cubic spline (RCS) analysis was conducted to ascertain the potential nonlinear associations between RDW or HRR and CA‐AKI. Multivariable log‐binomial regression models were fitted to assess the associations of RDW or HRR with CA‐AKI with the same covariates as linear regression models. Exploratory analysis in clinically relevant subgroups was conducted using fully adjusted log‐binomial regression models.

All *p* values were two‐sided and statistical significance was set at the level of 5%. Involved statistical analyses were all carried out using SPSS (version 25.0; SPSS Inc.) and R (version 4.0.5; R Foundation for Statistical Computing).

## 3 RESULT

### 3.1 Patient characteristics

After the eligibility screening, a total of 4054 patients who underwent either CAG or PCI were included in the study (Supporting Information: Figure S1). Table 1 provides an overview of the baseline characteristics of these enrolled patients. Patients in the CA‐AKI group were significantly older, more female, and were more prone to have diabetes (*p* < 0.05). RDW was higher in patients who developed CA‐AKI (13.9 [13.2, 14.9] vs. 13.5 [12.9, 14.2], *p* < 0.001), while HRR was lower (8.8 [7.3, 10.2] vs. 9.7 [8.6, 10.8], *p* < 0.001). Statistically significant differences between groups could also be observed in left ventricular ejection fraction, glycated hemoglobin A1c, hemoglobin, eGFR, and total cholesterol (*p* < 0.05). In terms of preadmission medications, fewer patients with CA‐AKI were treated with statin, aspirin, or clopidogrel (*p* < 0.001). However, there were no significant differences between groups in terms of hypertension, Scr at baseline, low‐density lipoprotein cholesterol, and angiographic and procedural features.

**Table 1:** Baseline characteristics of patients with/without CA‐AKI.

Characteristics	CA‐AKI		
	No (*n* = 3323)	Yes (*n* = 731)	*p* Value
Demographics			
Age, years	66.4 ± 10.7	69.2 ± 10.6	<0.001*
Female, *n* (%)	1097 (33.0)	292 (39.9)	<0.001*
BMI, kg/m^2^	24.3 ± 5.3	24.9 ± 5.3	0.018*
Clinical features			
Diabetes, *n* (%)	730 (22.0)	203 (27.8)	0.001*
Hypertension, *n* (%)	2065 (62.1)	469 (64.2)	0.328
LVEF, %	60.6 ± 12.6	56.9 ± 13.3	<0.001*
Laboratory measurements			
RDW, %	13.5 [12.9, 14.2]	13.9 [13.2, 14.9]	<0.001*
HRR	9.7 [8.6, 10.8]	8.8 [7.3, 10.2]	<0.001*
Scr, µmol/L	76.0 [64.0, 92.0]	73.0 [60.0, 97.0]	0.060
HbA1c, %	6.4 ± 1.3	6.7 ± 1.6	<0.001*
Hemoglobin, g/dL	13.0 ± 1.9	12.2 ± 2.3	<0.001*
eGFR, ml/min/1.73 m^2^	80.2 ± 21.5	76.6 ± 27.6	<0.001*
TC, mmol/L	4.16 ± 1.18	4.02 ± 1.22	0.004*
LDL‐C, mmol/L	2.25 ± 0.91	2.22 ± 0.90	0.392
Angiographic and procedural features			
Lesion location, *n* (%)			
LM	123 (3.7)	26 (3.6)	0.936
LAD	802 (24.1)	198 (27.1)	0.103
LCX	307 (9.2)	67 (9.2)	1
RCA	379 (11.4)	87 (11.9)	0.751
CTO, n (%)	159 (4.8)	41 (5.6)	0.403
Multivessel disease, n (%)	131 (3.9)	36 (4.9)	0.268
Direct PCI, n (%)	95 (2.9)	28 (3.8)	0.205
Volume of contrast agent, mg	70.00 [50.0, 130.0]	80.0 [50.0, 140.0]	0.144
Medication, *n* (%)			
Statin	2795 (84.1)	554 (75.8)	<0.001*
Aspirin	2816 (84.7)	524 (71.7)	<0.001*
Clopidogrel	2460 (74.0)	403 (55.1)	<0.001*

*Note*: Data are expressed as mean ± SD, median (interquartile range), or numbers (proportions).

Abbreviations: BMI, body mass index; CA‐AKI, contrast‐associated acute kidney injury; CTO, chronic total occlusion; eGFR, estimated glomerular filtration rate; HbA1c, glycated hemoglobin A1c; HRR, hemoglobin‐to‐red blood cell distribution width ratio; LAD, left anterior descending artery; LCX, left circumflex artery; LDL‐C, low‐density lipoprotein cholesterol; LM, left main coronary artery; LVEF, left ventricular ejection fraction; PCI, percutaneous coronary intervention; RCA, right coronary artery; RDW, red blood cell distribution width; Scr, serum creatinine; TC, total cholesterol.

* *p* < 0.05.

**Figure 1:**
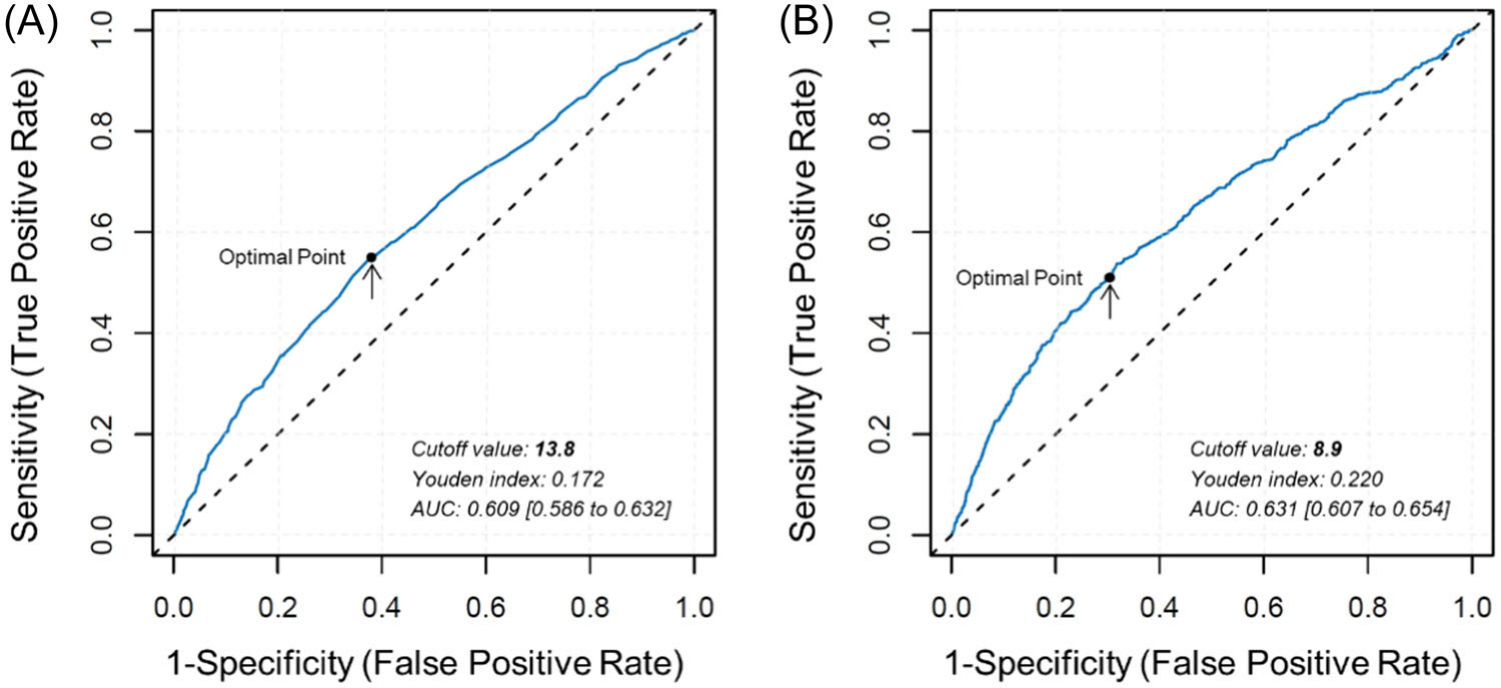
Receiver operating characteristic (ROC) analysis. The cutoff value of RDW (A) and HRR (B) for CA‐AKI was analyzed using ROC curves. The optimal cutoff value was evaluated according to the maximum Youden index and was pointed out with a dot and an arrow. AUCs were also calculated. AUC, area under the curve; CA‐AKI, contrast‐associated acute kidney injury; HRR, hemoglobin‐to‐red blood cell distribution width ratio; RDW, red blood cell distribution width.

The ROC curve analysis, using the Youden index, determined the cutoff values as 13.8% for RDW and 8.9 for HRR (Figure 1). A total of 1676 patients were subdivided into the high RDW group (RDW ≥ 13.8%), while the remaining 2378 patients were placed in the low RDW group (RDW < 13.8%). A total of 2605 patients were assigned to the high HRR group (HRR ≥ 8.9), with the remainder assigned to the low HRR group (HRR < 8.9).

### 3.2 Association between RDW and Scr elevation

**Figure 2:**
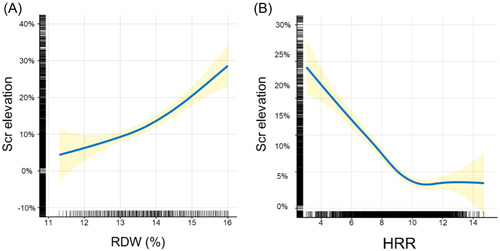
LOESS fit plot of RDW or HRR with Scr elevation. LOESS spline curve was plotted to estimate the association between RDW or HRR and Scr elevation. (A, B) The solid blue line represents the trend of Scr elevation with the increase of RDW or HRR, with a yellow shaded region around the curve indicating 95% CI. CI, confidence interval; HRR, hemoglobin‐to‐red blood cell distribution width ratio; LOESS, locally estimated scatter plot smoothing curve; RDW, red blood cell distribution width; Scr, serum creatinine.

Figure 2A illustrates the LOESS curve, providing a visual representation of the association between RDW and Scr elevation. As shown in the curve, an increase in RDW corresponds to a proportional increase in Scr elevation. Table 2 presents the results of univariable and multivariable linear regression models. These analyses demonstrate that RDW is significantly and positively associated with Scr elevation (unadjusted model: *β* = 3.149, 95% confidence interval [CI] = 2.449–3.848, *p* < 0.001; model 2: *β* = 2.496, 95% CI = 1.784–3.208, *p* < 0.001).

**Table 2:** Linear regression models of RDW or HRR with Scr elevation.

	Unadjusted model		Model 1		Model 2	
	*β* Coefficient [95% CI]	*p* Value	*β* Coefficient [95% CI]	*p* Value	*β* Coefficient [95% CI]	*p* Value
RDW	3.149 [2.449–3.848]	<0.001*	2.957 [2.257–3.657]	<0.001*	2.496 [1.784–3.208]	<0.001*
HRR	−3.702 [−4.305 to −3.099]	<0.001*	−3.596 [−4.249 to −2.943]	<0.001*	−3.559 [−4.243 to −2.875]	<0.001*

*Note*: Unadjusted model adjusted for none.

Model 1 adjusted for age (years), gender (female or male), diabetes (yes or no), and SBP (mmHg).

Model 2 additionally adjusted for LVEF (%), eGFR (mL/min/1.73 m^2^), excessive contrast medium (yes or no), and administration of statin (yes or no).

Abbreviations: CI, confidence interval; eGFR, estimated glomerular filtration rate; HRR, hemoglobin‐to‐red blood cell distribution width ratio; LVEF, left ventricular ejection fraction; RDW, red blood cell distribution width; SBP, systolic blood pressure; Scr, serum creatinine.

* *p* < 0.05.

### 3.3 Association between HRR and Scr elevation

LOESS curve in Figure 2B showed that with the increase of HRR, Scr elevation decreased sharply until HRR reached around 10.0, and then the curve became flat. In Table 2, linear regression models at the univariable and multivariable levels were performed. The results showed that HRR was strongly negatively associated with Scr elevation (unadjusted model: *β* = −3.702, 95% CI = −4.305 to −3.099, *p* < 0.001; model 2: *β* = −3.559, 95% CI = −4.243 to −2.875, *p* < 0.001).

### 3.4 Association between RDW and CA‐AKI

The curve generated by multivariable‐adjusted RCS analysis in Supporting Information: Figure S2A reveals a nonlinear association between RDW and CA‐AKI (*p* for nonlinearity = 0.0002). This indicates that as RDW increases, the risk of CA‐AKI rises in a nonlinear manner. In log‐binomial regression models, the low RDW group (RDW < 13.8%) was compared with the high RDW group (RDW ≥ 13.8%) as a reference group. The results in Table 3 demonstrate a strong association between high RDW (RDW ≥ 13.8%) and CA‐AKI (unadjusted model: relative risk [RR] = 1.763, 95% CI = 1.545–2.011, *p* < 0.001; model 2: RR = 1.540, 95% CI = 1.345–1.762, *p* < 0.001). The results presented in Supporting Information: Table S1 show that higher RDW levels (13.5%–13.9%, 14.0%–14.4%, and ≥14.5%) are also significantly associated with CA‐AKI (*p* < 0.05).

**Table 3:** Log‐binomial regression models of RDW or HRR binaries with CA‐AKI.

		Unadjusted model		Model 1		Model 2	
		RR [95% CI]	*p* Value	RR [95% CI]	*p* Value	RR [95% CI]	*p* Value
RDW	<13.8%	1 (Reference)		1 (Reference)		1 (Reference)	
	≥13.8%	1.763 [1.545–2.011]	<0.001*	1.665 [1.458–1.900]	<0.001*	1.540 [1.345–1.762]	<0.001*
HRR	≥8.9	1 (Reference)		1 (Reference)		1 (Reference)	
	<8.9	2.090 [1.835–2.382]	<0.001*	1.908 [1.663–2.189]	<0.001*	1.822 [1.584–2.096]	<0.001*

*Note*: Unadjusted model adjusted for none.

Model 1 adjusted for age (<75 or ≥75 years), gender (female or male), diabetes (yes or no), and SBP (<90, 90–119, 120–139, or ≥140 mmHg).

Model 2 additionally adjusted for LVEF (<40%, 40%–49%, or ≥50%), eGFR (<30, 30–59, 60–89, or ≥90 mL/min/1.73 m^2^), excessive contrast medium (yes or no), and administration of statin (yes or no).

Abbreviations: CA‐AKI, contrast‐associated acute kidney injury; CI, confidence interval; eGFR, estimated glomerular filtration rate; HRR, hemoglobin‐to‐red blood cell distribution width ratio; LVEF, left ventricular ejection fraction; RDW, red blood cell distribution width; RR, relative risk; SBP, systolic blood pressure.

* *p* < 0.05.

### 3.5 Association between HRR and CA‐AKI

Supporting Information: Figure S2B illustrates the results of the multivariable‐adjusted RCS analysis, which suggests a nonlinear association between HRR and CA‐AKI (*p* for nonlinearity = 0.0070). This nonlinearity indicates that as HRR increases, the risk of CA‐AKI decreases in a nonlinear fashion. Table 3 provides the outcomes of log‐binomial regression models revealing that low HRR (HRR < 8.9) is strongly associated with CA‐AKI (unadjusted model: RR = 2.090, 95% CI = 1.835–2.382, *p* < 0.001; model 2: RR = 1.822, 95% CI = 1.584–2.096, *p* < 0.001). Furthermore, HRR was assigned to five groups at equal intervals of 1.0 and the highest HRR group (HRR ≥ 11.0) was considered as the reference group. The results showed that lower HRR (<8.0 and 8.0–8.9) were still closely associated with CA‐AKI (*p* < 0.05) (Supporting Information: Table S2).

### 3.6 Exploratory analysis

Exploratory analysis, as depicted in Supporting Information: Figure S3, delves into the associations between RDW and HRR with CA‐AKI within various subgroups. Patients were stratified based on age (<75 or ≥75 years), gender (female or male), eGFR (<60 or ≥60 mL/min/1.73 m²), and anemia (yes or no). The findings across these subgroups consistently demonstrate that high RDW and low HRR remain strongly associated with CA‐AKI.

## 4 DISCUSSION

This retrospective cohort study has shed light on the associations of RDW and HRR with CA‐AKI in patients who underwent CAG or PCI. The key findings indicate that both high RDW and low HRR are significantly linked to CA‐AKI and represent independent risk factors for this condition.

CA‐AKI is a formidable complication following CAG or PCI, often contributing to prolonged hospitalization and adverse outcomes.^26^ Preventing CA‐AKI is of paramount importance since there is currently no definitive and effective treatment available.^26^, ^27^ Early identification of potential risk factors is the key to better CA‐AKI prevention.^28^ Current diagnosis of CA‐AKI mainly relies on clinical presentations of the urinary system and the levels of Scr elevation, but other indexes that could be for routine clinical use are comparatively limited.^22^, ^29^ Ideal indicators of CA‐AKI should be sensitive, cost‐effective, easily obtainable, and noninvasive.^6^ Laboratory variables can serve as much more meaningful indicators since the blood sampling test is one of the routine inspections for all admitted patients.

RDW is a measurement of the heterogeneity of circulating erythrocytes, which can be rapidly and easily obtained from blood routine tests.^20^ For decades, RDW has been mainly used to diagnose anemia.^30^ Recently, RDW is considered to be a novel biomarker for systemic inflammation and studies have proven that there existed a close association between RDW and CRP.^31^ In this regard, RDW is gaining widespread attention as an inflammatory indicator and a prognostic biomarker of cardiovascular diseases and renal diseases.^32^, ^33^ Lazzeroni et al. reported the potential of RDW to represent an independent predictor after myocardial revascularization or cardiac valve surgery.^34^ Akin et al. and Zhao et al. found that RDW was associated with the development of CA‐AKI in patients with ST‐segment elevation myocardial infarction and stable angina pectoris.^35^, ^36^ In accordance with previous studies, this study demonstrated that high RDW was an independent risk factor of CA‐AKI in all patients after CAG or PCI, indicating that enhanced surveillance of RDW might help to identify CA‐AKI earlier.

HRR, a composite indicator of hemoglobin and RDW, is a relatively new biomarker. Pooled evidence has shown that anemia was a significant risk factor for CA‐AKI.^37^ As an important diagnostic indicator for anemia, hemoglobin exerts its effectiveness in combining and delivering oxygen to tissues. Sreenivasan et al. demonstrated the role of decreased hemoglobin as a risk factor for CA‐AKI.^38^ Combing the hemoglobin and RDW, HRR was first proposed by Sun et al. and was found to be a powerful prognostic marker in patients with esophageal squamous cell carcinoma.^14^ Qu et al. also determined that low HRR was strongly associated with frailty in elderly patients with coronary heart diseases.^39^ However, there is no literature reporting the association between HRR and CA‐AKI. Based on the prognostic significance and biological properties of HRR, it is reasonable to believe that HRR may also be related to the risk of CA‐AKI and this study confirmed this conjecture. Anyway, since HRR is an emerging indicator, more research is needed to prove its value. The current study provided a basis for further comprehensive follow‐up studies.

Various factors act together to promote the occurrence of CA‐AKI, mainly including the toxicity of contrast medium, renal medullary ischemia, inflammation, and oxidative stress.^40^ However, the specific hypothesis to explain the association between RDW or HRR and CA‐AKI remains unclear. The proposed possible mechanism of CA‐AKI indicates that the injection of contrast medium can change renal hemodynamics and lead to hypoxia and ischemia in renal vessels, further resulting in the production of a large number of reactive oxygen species and the formation of oxidative stress.^41^, ^42^ The use of carvediol in patients with ACS can reduce the prevalence of CIN after urgent PCI, because carvedilol can inhibit inflammation, vasoconstriction, and oxidative stress.^43^ It is well‐established that oxidative stress and inflammation are intertwined, promoting each other.^44^ Both oxidative stress and inflammation can cause erythrocyte destruction and erythrocyte deformation, which can also be reflected through the change in RDW and HRR levels clinically.^45^, ^46^ Therefore, the above‐mentioned mechanisms may support our results about the associations of RDW and HRR with CA‐AKI.

It's important to acknowledge the limitations of this study, including its retrospective nature and potential selection bias. Additionally, the observational design only establishes associations and not causal relationships. Future large prospective and randomized controlled studies should further investigate these relationships while considering potential confounding factors.

## 5 CONCLUSION

The findings of this study underscore the clinical value of monitoring preoperative RDW and HRR in patients undergoing CAG or PCI. Both high RDW and low HRR demonstrated robust and independent associations with CA‐AKI. These findings suggest that tracking RDW and HRR could be valuable for the early identification of CA‐AKI risk.

## AUTHOR CONTRIBUTIONS

Wenbin Zhang and Xianglan Liu conceived and designed the study. Lijie Zhu and Zhezhe Chen organized these data and drafted the manuscript with the help of Hangpan Jiang, Peng Wang, and Maoning Lin. Hangpan Jiang analyzed the data. Hangpan Jiang and Lijie Zhu drew the pictures. Wenbin Zhang and Xianglan Liu detected any errors in the whole process. All authors have read and approved the manuscript for submission.

## CONFLICT OF INTEREST STATEMENT

The authors declare no conflict of interest.

## Supporting information

Supporting information.

## Data Availability

The datasets used and/or analyzed during the current study are available from the corresponding author upon reasonable request.
