# Supplementary material for: Association of red blood cell distribution width and hemoglobin‐to‐RDW ratio with contrast‐associated acute kidney injury in patients undergoing coronary angiography: A retrospective study
Source: Clin Cardiol. 2023 Oct 4;47(1):e24163. doi: 10.1002/clc.24163 (PMC10768739; doi:10.1002/clc.24163)
Supplement: Supplementary file 1 — Supporting information. [file CLC-47-e24163-s001.docx]

**Supplementary Material**

**Supplementary Tables**

**Table S1.** Log-binomial regression models of RDW (per 0.5%) with CA-AKI.

**Table S2.** Log-binomial regression models of HRR (per 1.0) with CI-AKI.

**Supplementary Figures**

**Figure S1.** Study flow chart.

**Figure S2.**Restricted cubic spline (RCS) analysis of RDW or HGG with CA-AKI.

**Figure S3.**Exploratory analysis.

**Table S1.** Log-binomial regression models of RDW (per 0.5%) with CA-AKI.

|  |  | Unadjusted model |  |  | Model 1 |  |  | Model 2 |  |
| --- | --- | --- | --- | --- | --- | --- | --- | --- | --- |
|  |  | RR [95% CI] | *P* value |  | RR [95% CI] | *P* value |  | RR [95% CI] | *P* value |
| **RDW** | <13.0% | 1 (Reference) |  |  | 1 (Reference) |  |  | 1 (Reference) |  |
|  | 13.0-13.4% | 1.117 [0.890 to 1.402] | 0.340 |  | 1.132 [0.903 to 1.420] | 0.282 |  | 1.108 [0.884 to 1.389] | 0.371 |
|  | 13.5-13.9% | 1.358 [1.085 to 1.700] | 0.007* |  | 1.326 [1.060 to 1.658] | 0.013* |  | 1.274 [1.020 to 1.593] | 0.033* |
|  | 14.0-14.4% | 1.582 [1.253 to 1.998] | <0.001* |  | 1.547 [1.227 to 1.951] | <0.001* |  | 1.451 [1.151 to 1.831] | 0.002* |
|  | ≥14.5% | 2.151 [1.771 to 2.612] | <0.001* |  | 1.985 [1.631 to 2.415] | <0.001* |  | 1.759 [1.438 to 2.151] | <0.001* |

Unadjusted model adjusted for none.

Model 1 adjusted for age (<75 or ≥75 years), gender (female or male), diabetes (yes or no) and SBP (<90, 90-119, 120-139, or ≥140 mmHg).

Model 2 additionally adjusted for LVEF (<40, 40-49, or ≥50%), eGFR (<30, 30-59, 60-89, or ≥90 ml/min/1.73m^2^), excessive contrast medium (yes or no) and administration of statin (yes or no).

CA-AKI: contrast-associated acute kidney injury; CI: confidence interval; eGFR: estimated glomerular filtration rate; LVEF: left ventricular ejection fraction; RDW: red blood cell distribution width; RR: relative risk. **P* <0.05.

**Table S2.** Log-binomial regression models of HRR (per 1.0) with CI-AKI.

|  |  | Unadjusted model |  |  | Model 1 |  |  | Model 2 |  |
| --- | --- | --- | --- | --- | --- | --- | --- | --- | --- |
|  |  | RR [95% CI] | *P* value |  | RR [95% CI] | *P* value |  | RR [95% CI] | *P* value |
| **HRR** | <8.0 | 2.716 [2.186 to 3.374] | <0.001* |  | 2.367 [1.881 to 2.979] | <0.001* |  | 2.245 [1.774 to 2.841] | <0.001* |
|  | 8.0-8.9 | 1.660 [1.300 to 2.119] | <0.001* |  | 1.481 [1.150 to 1.908] | 0.002* |  | 1.465 [1.138 to 1.885] | 0.003* |
|  | 9.0-9.9 | 1.194 [0.928 to 1.536] | 0.167 |  | 1.107 [0.858 to 1.430] | 0.434 |  | 1.099 [0.852 to 1.418] | 0.468 |
|  | 10.0-10.9 | 1.066 [0.823 to 1.380] | 0.628 |  | 1.022 [0.789 to 1.325] | 0.867 |  | 1.027 [0.793 to 1.330] | 0.840 |
|  | ≥11.0 | 1 (Reference) |  |  | 1 (Reference) |  |  | 1 (Reference) |  |

Unadjusted model adjusted for none.

Model 1 adjusted for age (<75 or ≥75 years), gender (female or male), diabetes (yes or no) and SBP (<90, 90-119, 120-139, or ≥140 mmHg).

Model 2 additionally adjusted for LVEF (<40, 40-49, or ≥50%), eGFR (<30, 30-59, 60-89, or ≥90 ml/min/1.73m^2^), excessive contrast medium (yes or no) and administration of statin (yes or no).

CA-AKI: contrast-associated acute kidney injury; CI: confidence interval; eGFR: estimated glomerular filtration rate; HRR: hemoglobin-to-red blood cell distribution width ratio; LVEF: left ventricular ejection fraction; RR: relative risk. **P* <0.05.

**Figure S1.** Study flow chart.


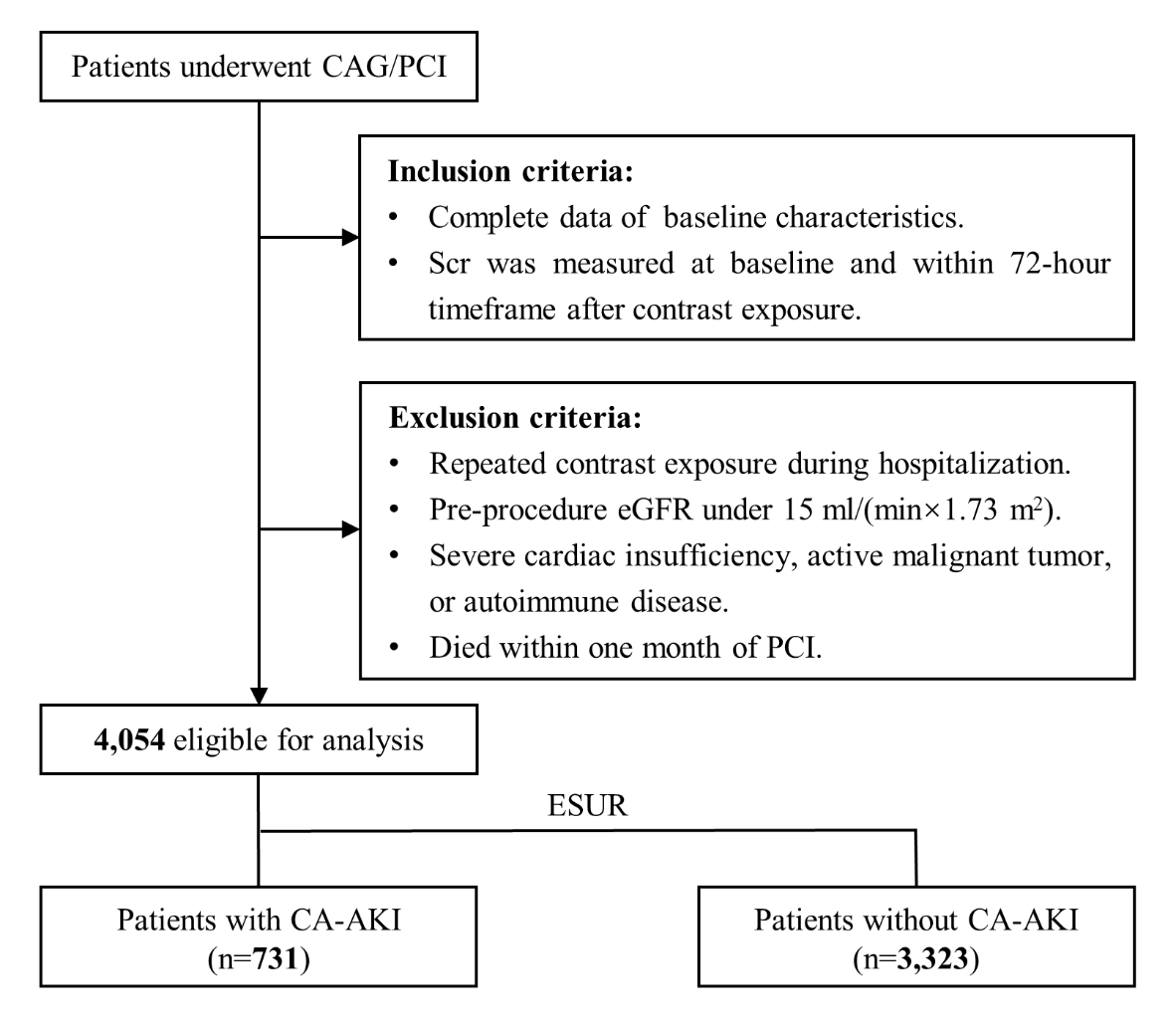


CA-AKI: contrast-associated acute kidney injury; CAG: coronary angiography; PCI: percutaneous coronary intervention; eGFR: estimated glomerular filtration rate; ESUR: European Society of Urogenital Radiology; Scr: serum creatinine.

**Figure S2.**Restricted cubic spline (RCS) analysis of RDW or HGG with CA-AKI.


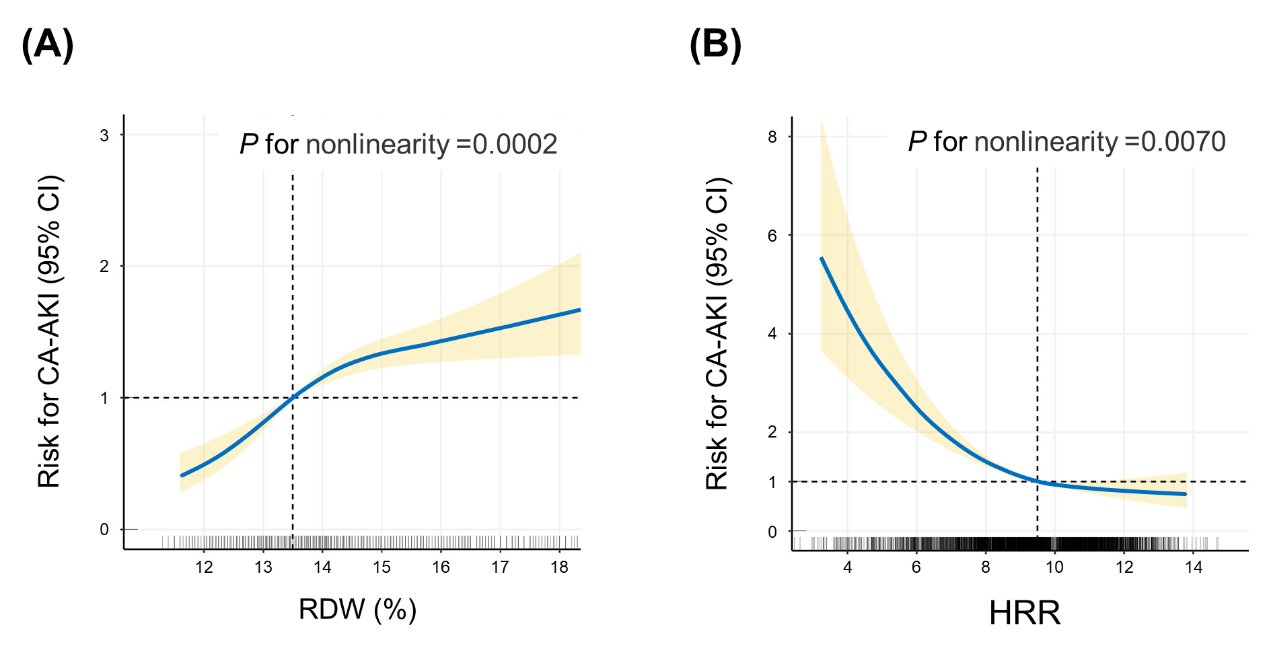


The potential nonlinear association between RDW (A) or HRR (B) and the risk of CA-AKI was evaluated using RCS analysis with multivariable adjustment. Four knots at the 5th, 35th, 65th, and 95th percentiles were chosen for flexibly modeling the association. Solid blue line represents the adjusted odds ratios, with yellow shadow around the curve indicating 95% CI.

CA-AKI: contrast-associated acute kidney injury; CI: confidence interval; HRR: hemoglobin-to-red blood cell distribution width ratio; RCS: restricted cubic spline; RDW: red blood cell distribution width.

**Figure S3** Exploratory analysis.

**
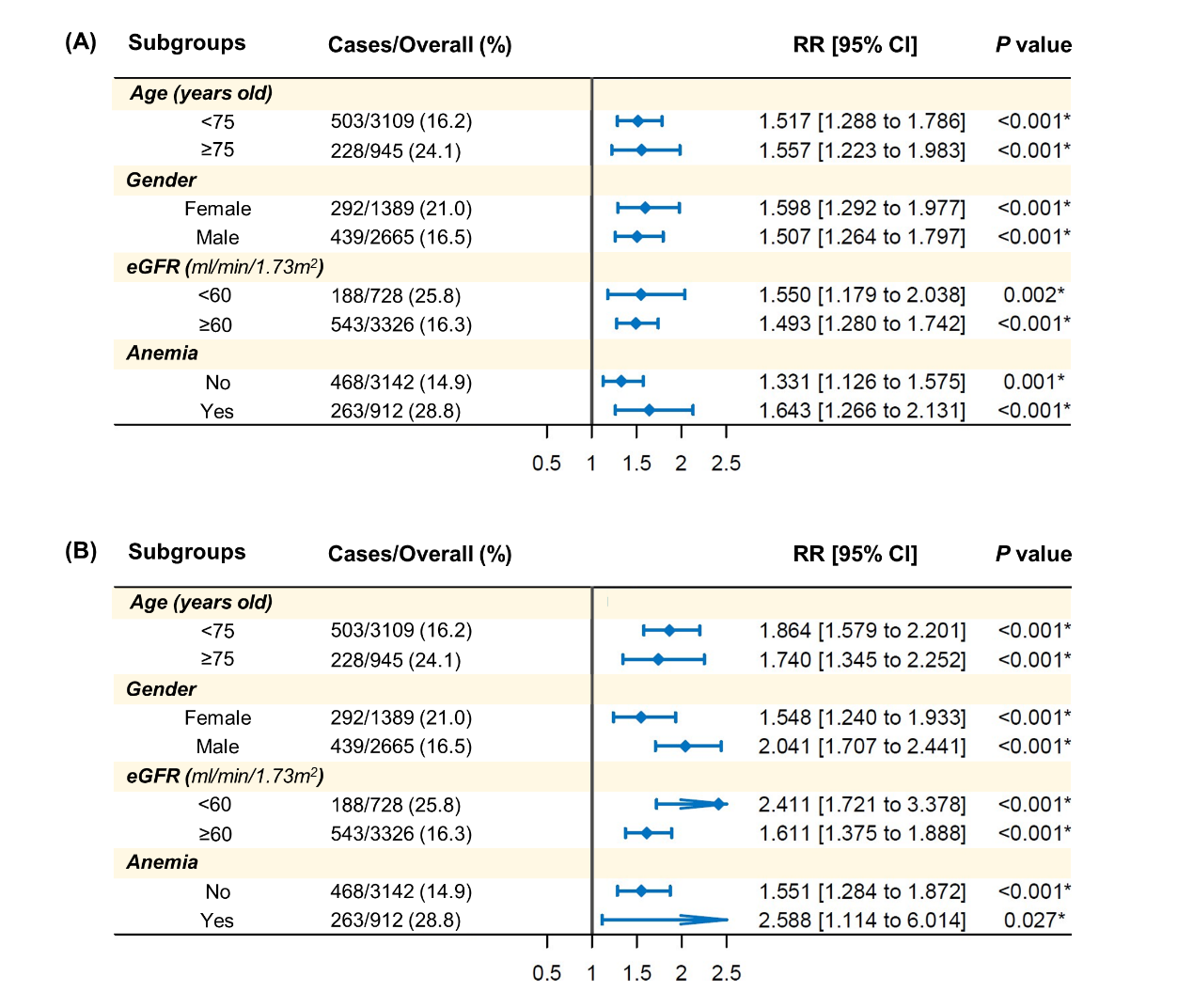
**

According to the stratification of age (<75 or ≥75 years old), gender (female or male), eGFR (<60 or ≥60 ml/min/1.73m^2^) and anemia (yes or no), log-binomial regression model was performed with the same covariates as model 2 in Table 3 to assess the effects of RDW or HRR on CA-AKI in different populations.

CA-AKI: contrast-associated acute kidney injury; CI: confidence interval; eGFR: estimated glomerular filtration rate; HRR: hemoglobin-to-red blood cell distribution width ratio; RDW: red blood cell distribution width; RR: relative risk. **P* <0.05.
